# Clinical significance of down-beating nystagmus and postural control loss when returning to a sitting position during the canalith repositioning maneuver

**DOI:** 10.1097/MD.0000000000032407

**Published:** 2022-12-30

**Authors:** Yee-Hyuk Kim

**Affiliations:** a Department of Otorhinolaryngology-Head & Neck Surgery, Daegu Catholic University School of Medicine, Daegu, Republic of Korea.

**Keywords:** benign paroxysmal positional vertigo (BPPV), falls, maneuver, nystagmus

## Abstract

Patients with benign paroxysmal positional vertigo (BPPV) occasionally experience severe dizziness, could not maintain the sitting posture, and then fall onto or off the examination table when they return to the sitting position, which is the last step of the barbecue maneuver and Epley maneuver (EM); down-beating nystagmus is also observed. This study aims to investigate the clinical characteristics and significance of these findings. We retrospectively reviewed video data showing nystagmus and medical records of adult patients diagnosed with canalolithiasis of the horizontal canal and the posterior canal (PC) BPPV who underwent barbecue maneuver and EM, respectively, in outpatient clinics from April 2014 to March 2019. This study included 112 patients (28 horizontal canal BPPV and 94 PC BPPV cases). Among the 122 BPPV cases, only 14 (14.9%) were analyzed, due to their occurrence during EM. Down-beating nystagmus appeared at 3.6 seconds on average after returning to the sitting position, and the patients fell onto or off the examination table at 4.4 seconds on average after the onset of the nystagmus. The average duration of the down-beating nystagmus was 20.3 seconds. In all 14 cases, no nystagmus was induced by the Dix–Hallpike test performed again after EM, confirming that the treatment was successful. During the EM, down-beating nystagmus and falling onto or off the examination table occurred in approximately 15% of cases. As the risk of falls increases, the patient should be secured immediately after EM. Moreover, it can be inferred that the findings occur when otoconia in the PC enter the utricle, suggesting a successful treatment.

## 1. Introduction

**Figure 1.**
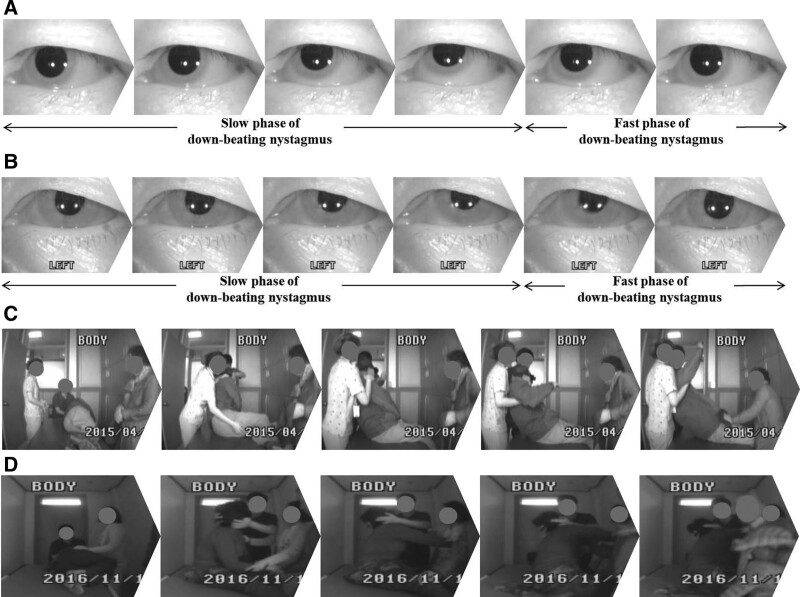
The series of images of patients with the findings of (A and B) down-beating nystagmus and (C and D) falling onto the examination table.

The canalith repositioning procedure (CRP) is the most suitable treatment for benign paroxysmal positional vertigo (BPPV), specifically the barbecue maneuver (BM) for horizontal canal (HC) BPPV and Epley maneuver (EM) for posterior canal (PC) BPPV.^[1]^ At times, patients returning to the sitting position, at the last stage of these 2 maneuvers,^[2]^ complain of severe dizziness and could not maintain their sitting posture, therefore eventually falling down; down-beating nystagmus also occurs (Fig. 1). To the best of our knowledge, 1 study reported this phenomenon.^[3]^ Therefore, the present study aims to discover the clinical characteristics and significance of the above findings in adult BPPV patients, in particular, with respect to the incidence rate, treatment outcomes, and the association of the down-beating nystagmus and postural instability. This study also intended to determine the latent time from the sitting position during CRP to the onset of down-beating nystagmus and falling onto or off the examination table, respectively; the duration time of the nystagmus; and the time period that the patient should be secured immediately after CRP to prevent falls.

## 2. Materials and methods

The medical records of 146 patients (168 cases) diagnosed with BPPV and underwent CRP at the outpatient clinic of the Department of Otolaryngology, Daegu Catholic University Medical Center in Korea, from April 2014 to March 2019, were retrospectively reviewed. The following were excluded: 1 case under 20 years of age, 10 cases that recurred on the same side within 3 months after diagnosis of BPPV, 4 cases with suspected BPPV of the anterior semicircular canal, 3 cases with suspected cupulolithiasis of the posterior semicircular canal, 21 cases diagnosed with cupulolithiasis of the horizontal semicircular canal, and 7 cases diagnosed as light cupula. All the remaining patients included in the study were treated by one and the same doctor.

The patients wore video Frenzel goggles (Easy-eyes®, SLMED, Seoul, Korea) during nystagmus tests, which included head roll test, bow and lean test, and Dix–Hallpike test, and during CRP.^[2,4]^ The BM and the EM were performed to treat HC BPPV and PC BPPV, respectively.^[2]^ To determine the success of the treatment after CRP, the patients rested in a sitting position for >20 minutes and then re-tested to confirm whether any evoked nystagmus, which occurs during nystagmus tests.^[5,6]^ Some patients underwent nystagmus tests 2 to 7 days after CRP to confirm that BPPV was well treated. In this study, video data showing nystagmus and medical records were used to identify the number of diagnosed patients by BPPV subtypes, the treatment success rate after CRP, the occurrence and latent time of falling onto or off the examination table, and latency and duration of down-beating nystagmus. The Institutional Review Board committee of the Daegu Catholic University Medical Center approved this study (CR-22-071).

## 3. Results

According to the inclusion/exclusion criteria, only patients with canalolithiasis type of HC BPPV and PC BPPV were included in this study. The medical data of 122 BPPV cases in 112 patients (50 men [54 BPPV cases]; 62 women [68 BPPV cases]; mean age, 59.6 ± 13.39 years; age range, 25–83 years) were analyzed in this study. In all 122 cases, 70 had lesions on the right and 52 on the left, and then 28 HC BPPV cases were subjected to the BM and 94 PC BPPV cases to the EM. Of 8 patients with 2 episodes of PC BPPV, 5 developed PC BPPV on the same side between 4 months and 3 years after the initial onset of PC BPPV, and 3 developed PC BPPV on the opposite side between 4 and 8 months after the initial onset of PC BPPV. Two patients initially developed HC BPPV and 1 year later developed PC BPPV. These suggest that the number of BPPV cases is 10 greater than the total number of patients, and the sum of the number of HC BPPV and PC BPPV patients is 2 greater than the total number of patients (Table 1).

**Table 1:** Summary of demographic and clinical data of all patients.

Number of patients	112 pt (122 BPPV cases)
Age	59.6 ± 13.39 yr
Sex	
Male	50 pt (54 BPPV cases)
Female	62 pt (68 BPPV cases)
Diagnosis	
HC BPPV-canalolithiasis	28 pt
PC BPPV-canalolithiasis	86 pt
Side of lesion	
Right	70 BPPV cases
Left	52 BPPV cases
Canalith repositioning procedure	
Barbecue maneuver	28 cases
Epley maneuver	94 cases

BPPV = benign paroxysmal positional vertigo, HC = horizontal semicircular canal, PC = posterior semicircular canal, pt = patients.

After CRP, the nystagmus test was performed again to determine the success of BPPV treatment: 100 cases after resting for >20 minutes, 8 cases on the second day, 3 cases on the third day, 4 cases on the fourth day, and 7 cases on the first week after CRP. When returning to the sitting position during EM, 14 patients with PC BPPV presented with down-beating nystagmus and postural control loss to fall onto or off the examination table after failing to maintain a sitting posture; this corresponds to 14.9%. However, none of the patients who performed the BM experienced this. During the EM, the average time until the onset of down-beating nystagmus after the patient returned to a sitting position was 3.6 ± 4.24 seconds (range: 1–18 seconds), and the average duration of down-beating nystagmus was 20.3 ± 7.46 seconds (range: 9–33 seconds). Falling onto or off the examination table after not keeping the body sitting posture, which means postural control loss in this study, appeared at an average of 8.1 ± 5.12 seconds (range: 2–21 seconds) after the patients returned to the sitting position during the EM. In other words, that is after an average of 4.4 ± 2.77 seconds (range: 1–10 seconds) after the onset of down-beating nystagmus (Table 2). All 14 patients rested in the sitting posture for >20 minutes after the EM, and then the Dix–Hallpike test was performed again on the same day, confirming that all of them did not develop evoked nystagmus by the test. It indicates the treatment was successful.

**Table 2:** Summary of demographic and clinical data of patients with down-beating nystagmus and falling onto or off the examination table when returning to a sitting position during canalith repositioning maneuver.

Number of subjects	14 patients (14 cases)
Age	67.9 ± 10.46 yr
Sex	
Male	6 patients
Female	8 patients
Canalith repositioning maneuver	
Barbecue maneuver	0 cases
Epley maneuver	14 cases
Lesion side	
Right	11 cases
Left	3 cases
Down-beating nystagmus	
Latency/range	3.6 ± 4.24 s/1–18 s
Falling onto or off the examination table	
Latency/range	8.1 ± 5.12 s/2–21 s
Down-beating nystagmus	
Duration/range	20.3 ± 7.46 s/9–33 s

Latency (mean ± standard deviation) refers to the time between returning to take a sitting position, the last stage of the Epley maneuver, and the occurrence of each finding.

## 4. Discussion

When the otoconia in the posterior semicircular canal enter the utricle by the EM, the inferior oblique muscle on the opposite side of the lesion is stimulated, and the superior rectus muscle on the ipsilateral side contracts, resulting in down-beating nystagmus.^[7–13]^ Therefore, if patients presented with down-beating nystagmus, it is highly likely that otoconia have entered the utricle, which can indicate that PC BPPV is treated well by the EM. Furthermore, at that time, the patients who complain of severe dizziness, could not control their body posture while sitting and sometimes fall onto or off the examination table. According to the patients in this study, dizziness at that time was not rotational, but a feeling of falling down or a feeling of the ground sinking like a vestibular drop attack. This may be caused by otoconia entering the utricle rather than by otoconia in the semicircular canal, which causes rotational vertigo. Therefore, if a patient shows down-beating nystagmus and falls onto or off the examination table when returning to the sitting position, it does not mean treatment failure but rather a predicting factor of successful treatment results. In fact, all 14 patients with the above findings were successfully treated for PC BPPV after the EM.

Another mechanism explains down-beating nystagmus that occurs when returning to the sitting position (fourth position) during the EM in the PC BPPV. When the otoconial mass of the posterior semicircular canal passes through the common crus and enters the utricle, it provokes aspiration of the endolymphatic column. To compensate for the depression of the space, an ampullofugal flow of endolymph occurs in the anterior semicircular canal. This acts as an excitatory stimulus to the anterior semicircular canal. Additionally, ampullopetal reflux of endolymph from the anterior semicircular canal to the posterior semicircular canal is induced. This acts as a weak inhibitory stimulus to the posterior semicircular canal.^[14,15]^ The occurrence of down-beating nystagmus in the fourth position of the EM is not uncommon. However, postural control loss implies falling onto or off the examination table without maintaining the body’s sitting posture, which is less frequent. It could be assumed that the size of the otoconial mass entering the utricle and the speed of the otoconial mass reaching the utricle affect postural control loss. Bilateral PC BPPV is a condition in which down-beating nystagmus could occur in the fourth position of the EM.^[16]^ When taking the fourth position of the EM in the bilateral PC BPPV, the otoconical mass in one of both posterior semicircular canals enters the utricle and creates ampullofugal flow in the ipsilateral anterior semicircular canal, and the otoconia in the opposite posterior semicircular canal move to the ampulla of the posterior semicircular canal and create an ampullopetal flow. These two phenomena may occur simultaneously and could cause intense down-beating nystagmus.

In the sitting position, the last step of the CRP, down-beating nystagmus, and falling onto or off the examination table were not observed in HC BPPV but only in PC BPPV. This is because the path of otoconia movement inside the utricle is thought to be different from HC BPPV and PC BPPV when the patients return to a sitting position during CRP. First, as the positions where the HC and the PC crura meet the utricle are different, in the HC BPPV and PC BPPV, the position of the otoconia in the semicircular canals will be different in the posture just before the last step of both CRPs. Therefore, the speed of the otoconia inside the utricle and the path of otoconia movements to the macula after otoconia enter the utricle will also be different when the patients return to the sitting position. Second, in the EM, otoconia in the PC enter the utricle when the patients return to the sitting position. However, in the BM, the otoconia in the HC may enter the utricle in a position where the body is lying on the side and the face is facing the floor just before returning to the sitting position.^[2]^ Therefore, severe dizziness may occur when the patients are seated at the last step of the EM in PC BPPV, which is severe enough to fall onto or off the examination table from a sitting posture. Although down-beating nystagmus did not occur while performing the BM in this study, in case of occurrence of a canal switch in which the otoconia enters the utricle at the beginning of the BM and then exits back into the posterior semicircular canal during the maneuver, down-beating nystagmus might occur when returning to the sitting position even in the BM.^[17]^

Hospital falls cause additional patient damage, prolonged hospital stays, and legal problems.^[18,19]^ Hospital falls were reported in 2.6 to 9.2 cases per 1000 patient days,^[20–23]^ which was estimated to correspond to 2 to 15% of inpatients,^[24]^ and 700,000 to 1000,000 cases annually in the US, of which 23 to 42% were injured by falls.^[20–22]^ Approximately 27.8% of cases of inpatient falls were patients who fell from the bed, and 10.2% were patients related to dizziness/vertigo.^[25]^ This indicates a high risk of falls in patients with severe dizziness/vertigo on the examination table, and it is imperative to prevent such falls during the EM. Nevertheless, although it is not uncommon for severe dizziness to occur when returning to the sitting position, little is known about it, and very few studies have been published.^[3]^ Thus, there will be some cases where preparations for falls are not thorough without considering these unexpected and dangerous situations that may occur during the EM. This study presented the latency of postural control loss, which means falling onto or off the examination table without maintaining the body sitting posture, and the latency and duration of down-beating nystagmus after returning to the sitting position during the EM. Therefore, if the EM is to be performed, it is necessary to refer to the data of this study and secure the patient firmly from immediately after the maneuver to the most prolonged latency period of the down-beating nystagmus in order to check whether the findings appear and prevent the patient from falls that may occur. This conforms to the concept of “nystagmus-based strategy” and “minimum stimulus strategy,” which means that moving to the next step after analyzing the variations of nystagmus appearing at each stage is necessary during CRP in a Frenzel video system environment.^[14–17]^

## 5. Conclusion

When some patients with PC BPPV returned to the sitting position, down-beating nystagmus appeared a few seconds later, and immediately after that, they felt severe dizziness and could not maintain the sitting posture and then fell onto or off the examination table. The patients who developed these findings have a high risk of falls, so it is necessary to secure the patient firmly immediately after the EM. These findings were observed in approximately 15% of patients who performed the EM. It can be inferred that this is a finding that occurs when the otoconia in the PC enter the utricle. Therefore, this phenomenon can be regarded as a finding suggesting a successful treatment result.
